# Robin Who? Bird Species Knowledge of German Adults

**DOI:** 10.3390/ani12172213

**Published:** 2022-08-28

**Authors:** Pirmin Enzensberger, Benjamin Schmid, Thomas Gerl, Volker Zahner

**Affiliations:** 1 Faculty of Forest Ecology and Management, University of Applied Sciences Weihenstephan-Triesdorf, 85354 Freising, Germany; 2 Institute for Biology Education, Ludwig-Maximilian University of Munich, 80797 München, Germany

**Keywords:** bird species knowledge, species identification skills, biological education, connectiveness with nature, representative survey

## Abstract

**Simple Summary:**

Biodiversity is declining worldwide, and knowledge of species and interest in nature are decreasing. The present study investigated the knowledge about bird species among the adult Bavarian population. Data were collected through a representative online survey with over one thousand respondents. The participants were asked to identify the species of birds they were shown pictures of. On average, 6 of the 15 species were identified correctly. Older participants scored higher than younger ones. The Eurasian blackbird showed the highest recognition rate, which correlates with the species abundance. Participants who performed better showed a higher tendency to act proactively for nature conservation, for instance donating money to NGOs.

**Abstract:**

Knowledge of species is the basis for involvement in biodiversity awareness and protection. For the first time, we investigated how bird species knowledge is spread among adults in Germany in a representative study. It was shown that of the 15 species presented, only 6 were recognized on average, and 4.5% of the tested persons did not recognize any species at all. Only 0.5% knew all presented species. Younger participants in particular knew significantly fewer species than the group over 60 years. We also tested if species knowledge has an impact on the motivation to act for nature conservation. In this study, knowledge of species correlated directly with the willingness to take action for species protection, e.g., through donating money for proactive nature conservation. Simply being in nature was meaningless for the test result. However, if one was actively involved with birds, e.g., via bird counts or bird feeding, species knowledge was significantly better.

## 1. Introduction

It is a common fact that the world’s ecosystems are within the process of a mass extinction of species [1,2]. One of the best indicators of biodiversity in an ecosystem is the species richness and abundance of birds [3]. Therefore, the population sizes of different bird species are quite well-known compared to other, less-studied taxa. Within the last decades, the biodiversity of birds declined rapidly [4,5]. A large-scale survey on the abundance of German birds reveals for example massive negative trends for species inhabiting farmlands and settlements since the 1990s [6]. In addition to the protection of species and their habitats, species knowledge is an important factor for saving biodiversity because taxonomic skills are necessary in all fields of conservational biology [7,8,9].

However, the observation and identification of birds not only contributes to a better understanding of ecosystems: it connects people to nature [10] with many benefits for personal well-being [11,12,13]. As birds are not very shy, the observation of birds is one of the rare occasions when people can interact with nature [14]. Feeding birds in urban gardens provides positive emotions [15] and therefore contributes to better connections to nature, which has been shown to be a significant predictor of personal happiness [16]. Some studies in environmental psychology show a trend that people prefer areas in urban environments with many birds [17,18], although it is not only the species richness but also the abundance of one single species that evokes positive emotions because many observers cannot distinguish between the different species [11]. An increased species knowledge of birds could therefore also lead to better personal well-being.

Prior surveys reveal that children do not know birds very well. Eight out of thirteen tested birds were practically unknown to Dutch primary school children, with identification rates under 6% [19]. The four least-known native vertebrates among German grammar school pupils were birds [20]. In surveys testing bird species knowledge among German children, they scored on average only one third of the possible points, with almost 30% of the pupils recognizing only three or fewer of the fifteen common bird species in the test [21]. Furthermore, the animal identification skill of German pupils has declined over the years. Today’s children could identify about 25% fewer birds [21] and 15% fewer vertebrates [20] than their peers in similar tests 10 years ago [22,23].

This decline in species knowledge over the years may lead to a shifting baseline syndrome [14] and should be stopped by educational means. To gain knowledge in general and species knowledge in particular, children need adult mediators who communicate this content to them [24,25]. Family members play a decisive role in this learning process [26], but by now, the few studies on the species knowledge of adults used randomly chosen test groups that might have been biased related to the selection of participants [19,26,27]. Our study presents results for the bird identification skills and factors influencing bird species knowledge within a group of German adults that is representative of the whole German population.

Species knowledge seems to be a key factor not only in conservation biology but also in the field of environmental psychology, but how many (bird) species can people identify? Recent studies have focused on the species knowledge of children and different factors influencing their ability to identify animal species [19,20,21,22]. These results were used to design our study on adults.

We hypothesized that bird species identification skills are age- and gender-dependent [19,20,21,22,28,29]. Based on previous research on children, we predicted that older people would have better identification skills than younger ones and that men would be able to identify more bird species than women because birders in Germany are mainly older males. In addition, we hypothesized that the number of inhabitants in a person’s hometown, or a person’s current place of residence, would have an impact on that person’s bird species knowledge [19,20,21,22,26]. We predicted that inhabitants of rural areas would score better than people in urban regions because they seem to have a closer contact to nature.

While other studies have been limited to socio-demographic aspects, we also wanted to examine for the first time how species knowledge affects conservation efforts. To do so, we hypothesized that participants’ donation behaviour and their membership in nature conservation organizations would influence their species knowledge. We predict that members of those NGOs score better in the identification test as well as those who donate money for nature conservation, because they are probably more interested in nature.

The active observation of birds should be taken into account as a predictor of species knowledge. We hypothesized that people who proactively observe birds have different bird species knowledge than others [8,19,20,21,22,30]. Our prediction is that both participants maintaining a bird feeding place and those who participate in citizen science projects like bird counts have higher species knowledge because they spend more time dealing with birds.

In addition, we analysed how various sources of species knowledge might influence the score on our identification test and determined the recognition rates of the tested species to find out how popular different species are among German adults.

## 2. Materials and Methods

We conducted an online survey with 1003 participants, randomly chosen from the ‘forsa.omninet’ panel. This panel is considered representative of the German-speaking population and consists of 80,000 participants, exclusively recruited by phone via multistage random selection (ADM master sample). Individuals without internet access will be provided a set-top box for the television to display visual content of surveys [31].

In the survey, the participants had to identify 15 different birds displayed in coloured pictures of these species, in an open-ended answer format. In addition to the species recognition test, the questionnaire included sociodemographic variables (age, gender, region, size of residence, political affiliation, household size and the number of children in the household) and items to explore the personal attitude of the participants towards nature conservation. To identify potential sources of species knowledge, participants were asked in what context they had learned about bird species in the past. To test whether their species knowledge was connected with their support of nature conservation, participants were asked whether they had donated to non-profit organizations in the past 12 months, distinguishing whether the purpose was conservation of nature and environment, animal welfare, or other.

The selection of the bird species and the photographic material taken by the authors were based on previous studies [21,22] that set a baseline for monitoring variations in the bird species knowledge of pupils over time. The selected species are listed in Table 1.

These common and abundant garden birds are very suitable for measuring species knowledge because they are rather easy to observe and to distinguish with visible characteristics. In addition, abundant data from citizen science projects like the German Bird count are available.

With the exception of three, this list consists of the 15 bird species with the most observations in the German winter bird count [32]. As there are two similar sparrow species (*Passer montanus* and *P. domesticus*) among these frequently observed species, both named “sparrow” in German, we chose the house sparrow to diminish confusion. Carrion crows (*Corvus corone*) are also seen very often in gardens, but they are hard to distinguish from common ravens (*Corvus corax*), as their size is not properly visible in pictures. Therefore, we substituted this species by the Eurasian Siskin (*Spinus spinus*). The common wood pigeon (*Columba palumbus*) was replaced by the northern wren (*Troglodytes troglodytes*).

To quantify the species knowledge, we used a previously tested method [20,21,22]. Participants received 1.0 point per recognized species and 0.5 point per recognized superior taxonomic group, allowing participants to reach a maximum of 15 points. If the participants named the wrong species of the correct superior taxonomic group, they also received 0.5 point. If a participant, e. g., mixed up the blue tit with a great tit, the participant received 0.5 point for the superior taxonomic group but not the full score because the species was not correctly identified.

The sum of the points scored served as a measure of personal species knowledge. The reliability of the scale was calculated with Cronbach’s alpha. The value α = 0.88 indicates a high internal consistency of the scale [33]. 

To test the hypotheses, both difference and correlation analyses were performed. Since the hypothesis of normal distribution of the variable species knowledge was disproved by the Shapiro–Wilk test (W (1003) = 0.98, *p* < 0.05) and by the Kolmogorov–Smirnov test (D (1003) = 0.34, *p* < 0.05), only nonparametric tests were conducted to test statistical significance. 

Therefore, the Wilcoxon rank-sum test was used to determine significance of the rank-sum differences of two groups. For more than two groups, the Kruskal–Wallis test and pairwise analysis of rank-sum differences was performed, using the pairwise Wilcoxon rank-sum test with Bonferroni correction to counteract the multiple-comparisons problem. The threshold for significance was chosen to be *p* = 0.05. To determine the size of the difference between two medians, the effect size r was calculated. In the following, this is denoted by r_eff_ to distinguish it from the correlation coefficient. To examine correlations between the recognition rate of a species and its abundance, the Spearman correlation coefficient r was calculated.

Statistical analysis was conducted in RStudio 1.3.959 [34] in particular using the psych [35] and dplyr [36] packages.

Recognition rates were used to compare the popularity of the different bird species. According to previous studies, the recognition rate is the sum of scores for the species divided by the number of participants [20]. Therefore, it combines recognition on species at higher taxonomic levels. The higher this recognition rate, the better known the bird is within the test group (Table 2).

To investigate the reasons for the different recognition rates of the species, Spearman correlation analyses were performed. Therefore, data on the abundances of the individual species were required. The percentages of Bavarian gardens in which a species was counted during the citizen-science projects garden bird count (“Stunde der Gartenvögel 2020”) and winter bird count (“Stunde der Wintervögel 2021”) were used to define the frequency of occurrence during summer as well as winter [32,37].

## 3. Results

The participants achieved an average of 6.89 (SD = 3.69) points, which corresponds to just under 46% of the maximum possible score of 15 points. Among the participants, 4.5% could not correctly identify a single bird species, and only 0.5% of the 1003 participants were able to correctly name all 15 species presented.

### 3.1. Sociodemographic Factors

The performed Kruskal–Wallis tests revealed significant differences in the species knowledge for different age groups (χ^2^ = 52.097, df = 3, *p* < 0.001) and the number of inhabitants in the current place of residence (χ^2^ = 9.5758, df = 3, *p* < 0.05) as well as the number of inhabitants in the hometown (χ^2^ = 9.2291, df = 3, *p* < 0.05).

**Figure 1:**
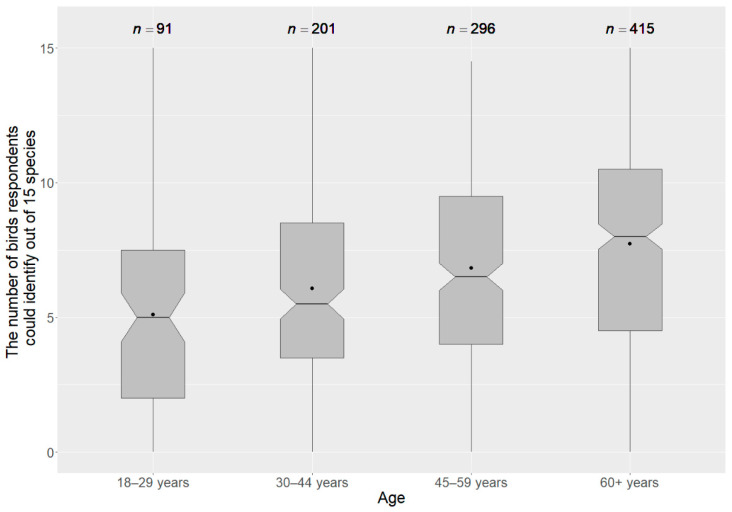
Bird species knowledge by participants of different age groups. Lower end of the boxplot shows the first quartile, the median is indicated by a line in the notch (95% confidence interval of the median) and the 75% quartile is marked by the upper end of the boxplot. The dot within the boxplot indicates the mean score.

In our test, older participants scored better on the bird identification test (Figure 1). The pairwise Wilcoxon rank-sum test showed that participants over 60 years performed significantly better than all other groups. They performed better than the group of 18- to 29-year-olds (W = 11,426, *p* < 0.001, r_eff_ = 0.24), the 30- to 44-year-olds (W = 30,939, *p* < 0.001, r_eff_ = 0.19), and the 45- to 59-year-olds (W = 52,668, *p* < 0.05, r_eff_ = 0.09). The 45- to 59-year-old participants also performed significantly better than the 18- to 29-year-olds (W = 9731.5, *p* < 0.001, r_eff_ = 0.17).

Our results show a decrease in species knowledge with an increasing number of inhabitants in the current place of residence. However, the post hoc pairwise Wilcoxon rank-sum comparison showed only significant differences between participants from cities with over 100,000 inhabitants (M = 6.39, SD = 3.68) and those from places with under 5000 inhabitants (M = 7.32, SD = 3.73, W = 29,866, *p* = 0.045, r_eff_ = 0.05).

The pairwise Wilcoxon rank-sum comparison between groups with different numbers of inhabitants in their hometowns showed significant differences between people from cities with 20,000–100,000 inhabitants (M = 6.43, SD = 3.62) and those from places with less than 5000 inhabitants (M = 7.32, SD = 3.65, W = 35,063, *p* = 0.03, r_eff_ = 0.059).

For the entirety of the respondents, no significant difference could be found between women and men. The education level of the participants as well as their political convictions had no significant effect on their performance in the test.

### 3.2. Sources of Species Knowledge

**Figure 2:**
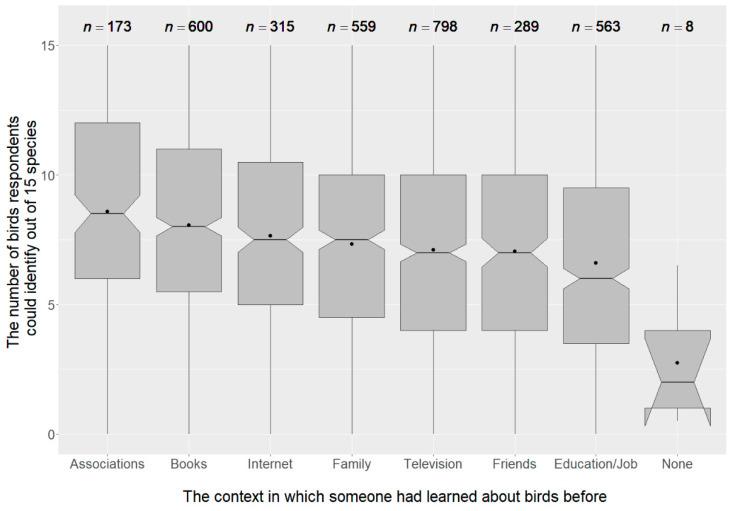
Bird species knowledge on the identification test depending on the specified source of knowledge. Lower end of the boxplot shows the first quartile, the median is indicated by a line in the notch (95% confidence interval of the median) and the 75% quartile is marked by the upper end of the boxplot. The dot within the boxplot indicates the mean score.

Bird species knowledge varied with the participants’ chosen sources of information (Figure 2).

The highest score, with an average of 8.59 (SD = 3.9) points was achieved by participants who stated that they had learned about birds through nature conservation organisations (*n* = 173). Even though more than half of the participants (*n* = 563) reported that they had gained knowledge about birds in the context of their job or education, this source achieved the lowest score with an average of 6.6 (SD = 3.63) points and thus counts as a relatively inferior source of species knowledge. The fact that knowledge acquired through nature conservation organisations resulted in the highest species knowledge was also evident in other results shown in the following paragraphs.

### 3.3. Species Knowledge and Attitude towards Nature Conservation

**Figure 3:**
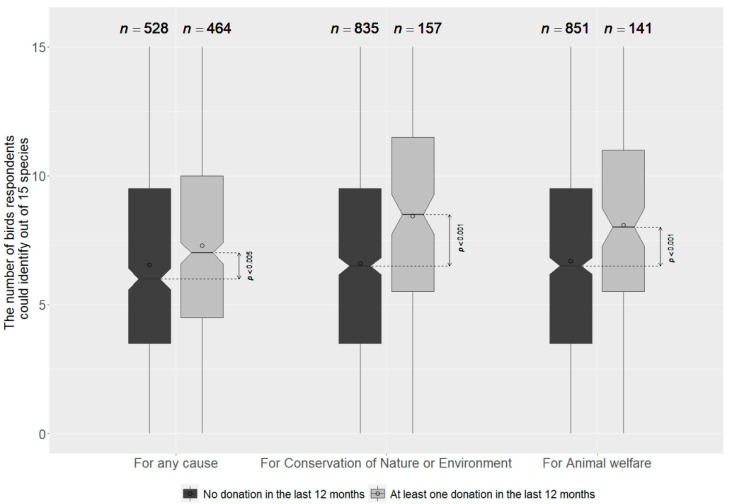
Bird species knowledge of donors (grey) compared with non-donors (black) with significance level p and the number of participants in the group n. Lower end of the boxplot shows the first quartile, the median is indicated by a line in the notch (95% confidence interval of the median) and the 75% quartile is marked by the upper end of the boxplot. The dot within the boxplot indicates the mean value of the score.

Participants who stated to be members of nature conservation organisations performed better than non-members. The Wilcoxon rank sum test showed a significant difference between these groups (W = 93,169, *p* < 0.001, r_eff_ = 0.26). Donors to non-profit organisations were found to have significantly higher species knowledge than non-donors (W = 144,570, *p* < 0.001, r_eff_ = 0.1). This was particularly true for people who said they had donated for nature conservation (W = 47,053, *p* < 0.001, r_eff_ = 0.17) or animal welfare (W = 47,053, *p* < 0.001, r_eff_ = 0.13) (Figure 3).

Participants who fed birds scored significantly higher on the identification test (χ^2^ = 51.717, df = 2, *p* < 0.001). Participants who reported feeding birds year-round had the highest result with an average of 8.46 (SD = 3.49) points. They performed significantly better than participants who fed birds exclusively in winter (M = 7.36, SD = 3.6, W = 32,143, *p* < 0.001, r_eff_ = 0.13) and those who never fed birds (M = 5.68, SD = 3.43, W = 8956.5, *p* < 0.001, r_eff_ = 0.36). The difference between feeding in winter and not feeding at all was also significant (W = 25,750, *p* < 0.001, r_eff_ = 0.2). Participation in Citizen Science projects was also related to higher species knowledge. People who had participated multiple times in a garden or winter bird count achieved significantly higher scores (M = 10.66, SD = 3.23) than people who had not yet participated (M = 6.55, SD = 3.57, W = 11,140, *p* < 0.001, r_eff_ = 0.24). Participation also resulted in a significantly higher score (M = 9.22, SD = 3.01) compared with non-participation (W = 5995, *p* < 0.001, r_eff_ = 0.13).

### 3.4. Popularity of Species in Comparison

**Figure 4:**
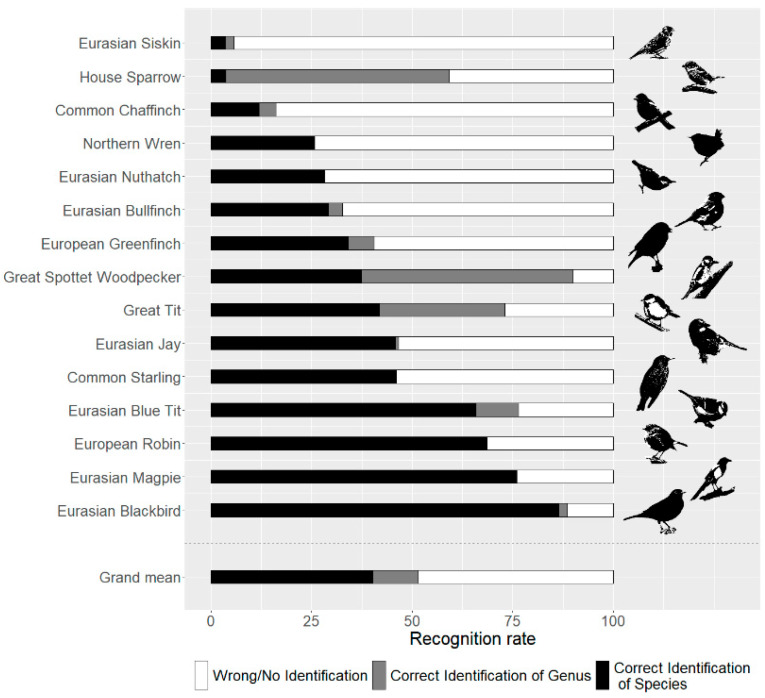
Species sorted by their recognition rates on species level.

The recognition rates varied greatly between the species (Figure 4). At the species level, only the Eurasian blackbird was well-known, and three others (Eurasian magpie, European robin, Eurasian blue tit) were rather well-known. Eight of the remaining eleven species were rather unknown, and three (common chaffinch, house sparrow, Eurasian siskin) three were entirely unknown to the participants.

Five of the fifteen species were either identified at the species level or completely unknown. For the remaining ten, some proportions of the participants could identify these birds at the superior taxonomic level. For the house sparrow and the great spotted woodpecker, at the higher taxonomic level, the answers “sparrow” and “woodpecker”, respectively, contributed more than half of the score to total recognition rate, while almost one third of all participants named the great tit just “tit”.

Twelve of the fifteen bird species on the test had higher recognition rates within the group of adults compared with the similar survey with pupils [21]. Three species (Eurasian Siskin, northern wren and house sparrow) were more popular among children (Table 3).

Regarding the Spearman’s correlation analyses, the recognition rate was strongly correlated with the frequency of occurrence in gardens (p_winter_ = 0.01631, r_winter_ = 0.62, N_winter_ = 15; p_summer_ = 0.005492, r_summer_ = 0.69, N_summer_ = 15), with individual species deviating from this correlation (Table 3). An example for this is the chaffinch, which is one of the most common garden birds in Germany but had one of the lowest recognition rates.

## 4. Discussion

In a situation where the shrinking of species knowledge runs parallel to the decline in species abundance and species richness, along with numerous factors like loss of biodiversity and well-being, the question arises of what are the factors that enhance species knowledge.

### 4.1. Sociodemographic Factors

Concerning the parameters tested in our survey, species knowledge increases with age. In almost all other studies, age was also a significant predictor of species knowledge [38], but species knowledge does not simply increase with life experience; it also decreases when comparing age cohorts of different times. In the year 2007, German pupils scored 15% more points on a same-species identification test than they did in a follow-up study a decade later [20,21], for multiple possible reasons. Less training on birds due to changes in curricula [21] could be a reason for these differences between younger and older participants. Young adults up to age 25 years with a degree in higher secondary education were not taught in bird species during their time in grammar school due to the prevailing curriculum. Older participants with the same educational degree were educated in ornithology because it was mandatory in their curriculum. Nevertheless, these lessons in school are long ago, and probably much of their content is forgotten. Therefore, other explanations like changes in avian biodiversity [4,5], less observability in the living environment, or more interest in a more virtual world [22] should be taken into account to explain the changes in bird species knowledge along with age.

The hypothesis that people from rural areas should have better species knowledge, is supported in this study. This is in line with other studies [39]. Nevertheless, the small difference in species knowledge between the inhabitants of rural and urban areas is rather surprising, since several surveys summarized in [40] report a strong disconnection between urban citizens and nature. This disconnection seems to have only a small influence on bird species knowledge as other surveys observed even slightly better bird species knowledge in the group of urban citizens [21] or no significant influence of the hometown’s number of inhabitants at all [19,20]. Another study found hometown sizes not relevant, but a distance >10 km to the next forest patch was correlated with a lower identification score [38]. Although we did not test the species knowledge of different indigenous groups, our results might support the presumption that members of post-industrial societies have less knowledge of nature than members of indigenous communities [41]. Further research on this topic might be helpful for clarifying this open question.

Previous studies confirm that a connection to nature is mainly formed in childhood [21,22,39]. Our results indicate that it is not the size of the hometown where people grew up that makes the difference but a different factor (like, e.g., influence of relatives) that has yet to be identified in future studies.

Nevertheless, this study is the first presentation of baseline bird species knowledge among a representative population of German adults. Further investigations on the effectiveness of educational projects to foster species knowledge could be evaluated in a pre-/posttest design using our data as a reference. This could show how bird species knowledge is evolving in the future.

### 4.2. Knowledge and Connectiveness with Nature

Our study showed that people with higher scores on our species knowledge test were more active in citizen science projects and in nature conservation organizations and vice versa. The fact that repeated participation in citizen science projects resulted in the highest scores suggests that participation leads to a learning effect in terms of species knowledge or vice versa: That is, people with higher test results, i.e., a better knowledge of bird biodiversity, might have had greater willingness to act proactively for nature conservation such as donating money to an NGO. The higher knowledge of species among donors could be due to a comparatively high interest in nature and animal protection-related issues. This knowledge motivates people to protect nature by donating money for its conservation. However, the results also show that donating increases with age.

A representative 2019 study about nature awareness in Germany took a closer look at social milieus and species knowledge [42]. Members of the nature-loving milieus of the liberal-intellectuals and social-ecologicals had the best species knowledge. These milieus also show a high willingness to preserve biological biodiversity. This is an indication of the strong interaction between connectiveness with nature, species knowledge and attitude towards nature conservation.

However, species knowledge is not important only for nature conservation efforts. Studies show that species knowledge is a key factor for personal well-being [11,12]. Bird related activities could therefore enhance the quality of life.

### 4.3. Sources of Species Knowledge

The points above highlight the importance of species knowledge for natural research, conservation and personal well-being, but how is species knowledge formed and acquired?

Comparing different sources of species knowledge showed large differences. NGO educational programs seemed to have the highest effect regarding providing species knowledge, while education and job environments ranked last. However, while educational programs offered by NGOs are voluntary and more likely to be taken up by people interested in nature, schooling is not. This could also explain the differences in the resulting scores.

Species knowledge develops best in an experiential and interactive way [21,22,43]. The comparison of a teacher-centred form of instruction and a form of instruction in which pupils worked in small groups with bird specimens, which resembles the environmental education offered by NGOs, showed that the group that actively engaged with the birds had higher species knowledge.

As the basis of species knowledge is laid during childhood, schools should be a key factor. However, formal education procedures performed poorly in our study. Other authors suspect curriculum changes, such as the missing thematization of native birds, as a reason for the decline in species knowledge [21]. The changing orientation of universities toward a focus on molecular biology and the accompanying reduction of taxonomic chairs could also be a reason why formal education processes are not very successful in teaching species knowledge [43]. Therefore, self-learning processes should be initiated to improve species knowledge, especially within the group of adults who are no longer part of the educational system [44].

But how do adults learn about birds? The most frequent mentions of television and books as the origin of species knowledge differ substantially in their ability to impart species knowledge. Following the ICAP model explaining the effectiveness of different teaching methods [45], it can be assumed that a mere passive consumption of bird-related content like in TV broadcasts leads to only small gains in species knowledge. While the consumption of books represents a more active action, the knowledge gain is higher than in passive learning processes. However, books dealing with birds are also more likely to be consumed by people already interested in the subject, whereas television programs such as nature documentaries probably appeal to a wider audience.

The internet ranked third in its ability to impart species knowledge. It offers numerous platforms and identification aids for beginners, advanced users and specialists with regard to species knowledge and is expected to show increasing influence as a source of information for those interested. There is a wide range of different online materials from more or less passive tools that range from written books to explanatory videos similar to TV broadcasts, but with a special focus on bird-related content. Most recently, the online teaching community has also developed learning materials that engage the learners in a constructive or even interactive way [46,47,48]. The users have to fulfil certain tasks in an online connected group and obtain corrected feedback similar to formal education processes. As this kind of teaching is most effective [45], efforts should be made to promote these platforms. Further investigations should also clarify which teaching materials are most suitable for adults according to their effectiveness, acceptance and availability.

### 4.4. Popularity of Species

Birds were quite unknown to the adult participants in this survey. This is in line with findings from previous studies indicating that birds are the least-known group of vertebrates among German pupils [20]. These findings are quite surprising, as birds are—compared with other vertebrates—rather easy to observe due to their diurnal activities and their comparatively small flight distances. In addition, their agility and their colourful plumage are fascinating to watch. However, our results show that outside of the group of “birders”, most species are rather unknown or even unknown to the majority of citizens.

To explain the different recognition rates of species is not an easy task. One key factor seems to be the frequency of observing the species: The more frequently the bird appears near human settlements, e.g., in gardens, the higher its recognition rate, although there are exceptions to this phenomenon. One of the most abundant German birds, the chaffinch, was unknown to more than three quarters of the participants: That is, almost everyone has seen a chaffinch, but only a few people can recognize and name it properly.

There are probably additional factors influencing the recognition rates, e.g., the morphology of the bird. The four biggest birds in the test are among the five species with the highest recognition rates, but size does not seem to be the only morphological characteristic influencing the species’ recognition rates. Species with eye-catching colours are more popular than inconspicuous birds. Featherings with rich contrasts seem to foster the ability to recognize the bird. Good examples are the black body with eye-catching yellow beak of the blackbird, the black and white colours of the magpie or the iridescent shining of the starling. The number of different colours in the plumage seems to be less important in explaining the popularity of species, as there are colourful birds among the rather well-known species (e.g., great and blue tit) as well as among the (rather) unknown birds (chaffinch, nuthatch, siskin).

In previous surveys on German pupils, there was a correlation between the German species name and the popularity of the bird [49]. Our test supports the hypothesis that species with names referring to morphological characteristics are easier to recognize than others. The German names for the robin and the blue tit include the colour of the plumage, and both species are rather well-known. On the other hand, the greenfinch does not fit this model, as its name contains a morphological characteristic, but the species is actually rather unknown.

The significant difference in the recognition rates for the great spotted woodpecker versus the house sparrow at the species level and at a superior taxonomic level lead to a linguistic explanation for why some species are more popular than others: For these two birds, the participants in our test used the classification for the family because this is also the name of these birds in everyday language. The participants were probably not aware that there was more than one species in this superior taxonomic group. They did not know that there is a field sparrow in addition to the house sparrow or several quite similar woodpeckers in addition to the one in our test.

Therefore, species knowledge is more than knowing the name of the bird. It is necessary to know about the existence of other similar species in order to distinguish them properly. Further investigations should be conducted to verify this hypothesis using individuals of the same species with different looks (old and young, male and female) and different species that are quite similar.

## 5. Conclusions

We tested a representative sample of adults in Germany for the first time. These data could be an important baseline for evaluating the success of a wide range of educational strategies to improve bird species knowledge. This makes it possible to filter the results for crucial predictors like age, the source of species knowledge and the conspicuousness of species. We showed that people with higher species knowledge had stronger connectiveness with nature and were more willing to act proactively for nature conservation.

## Figures and Tables

**Table 1:** List of bird species tested in this study.

Great Tit (*Parus major*)	Eurasian Blackbird (*Turdus merula*)
Eurasian Magpie (*Pica pica*)	House Sparrow (*Passer domesticus*)
Eurasian Bullfinch (*Pyrrhula pyrrhula*)	Common Chaffinch (*Fringilla coelebs*)
Great Spotted Woodpecker (*Dendrocopos major*)	Eurasian Nuthatch (*Sitta europaea*)
European Greenfinch (*Chloris chloris*)	Northern Wren (*Troglodytes troglodytes*)
European Robin (*Erithacus rubecula*)	Common Starling (*Sturnus vulgaris*)
Eurasian Siskin (*Spinus spinus*)	Eurasian Blue Tit (*Cyanistes caeruleus)*
European Jay (*Garrulus glandarius)*	

**Table 2:** Different categories of popularity defined by the recognition rates of the bird species.

Recognition Rate	Popularity of Species
100–75%	Well-known
74–50%	Rather well-known
49–25%	Rather unknown
24–0%	Unknown

**Table 3:** Recognition rates within the group of pupils and adults in comparison with the frequency of occurrence in gardens measured as the number of observations during the Citizen Science projects winter bird count 2021 and garden bird count 2020. Data from [21,32,37].

	Recognition Rate		Frequency of Occurrence in Gardens	
Species	Pupils [21]	Adults	Winter [32]	Summer [37]
Eurasian Blackbird	78%	87%	86%	94%
Eurasian Magpie	62%	76%	45%	55%
Eurasian Blue Tit	64%	71%	71%	63%
Eurasian Robin	67%	69%	58%	42%
Great Spotted Woodpecker	61%	64%	36%	28%
Great Tit	44%	55%	84%	81%
Eurasian Jay	36%	46%	14%	19%
Common Starling	32%	46%	2%	58%
European greenfinch	18%	37%	29%	37%
House Sparrow	39%	31%	54%	64%
Eurasian Bullfinch	21%	31%	8%	7%
Eurasian Nuthatch	24%	28%	28%	17%
Northern Wren	30%	26%	8%	5%
Common Chaffinch	14%	14%	44%	33%
Eurasian Siskin	11%	5%	19%	2%

## Data Availability

The data presented in this study are available on the Open Science Framework at https://osf.io/j24bs/files/ (Accessed on 21 August 2022).
